# Machine learning compensates fold-change method and highlights oxidative phosphorylation in the brain transcriptome of Alzheimer’s disease

**DOI:** 10.1038/s41598-021-93085-z

**Published:** 2021-07-01

**Authors:** Jack Cheng, Hsin-Ping Liu, Wei-Yong Lin, Fuu-Jen Tsai

**Affiliations:** 1 Graduate Institute of Integrated Medicine, College of Chinese Medicine, China Medical University, Taichung, 40402 Taiwan; 2 Department of Medical Research, China Medical University Hospital, Taichung, 40447 Taiwan; 3 Graduate Institute of Acupuncture Science, College of Chinese Medicine, China Medical University, Taichung, 40402 Taiwan; 4 Brain Diseases Research Center, China Medical University, Taichung, 40402 Taiwan; 5 School of Chinese Medicine, China Medical University, Taichung, 40402 Taiwan; 6 Department of Medical Laboratory and Biotechnology, Asia University, Taichung, 41354 Taiwan; 7 Division of Pediatric Genetics, Children’s Hospital of China Medical University, Taichung, 40447 Taiwan

**Keywords:** Machine learning, Neurological disorders

## Abstract

Alzheimer’s disease (AD) is a neurodegenerative disorder causing 70% of dementia cases. However, the mechanism of disease development is still elusive. Despite the availability of a wide range of biological data, a comprehensive understanding of AD's mechanism from machine learning (ML) is so far unrealized, majorly due to the lack of needed data density. To harness the AD mechanism's knowledge from the expression profiles of postmortem prefrontal cortex samples of 310 AD and 157 controls, we used seven predictive operators or combinations of RapidMiner Studio operators to establish predictive models from the input matrix and to assign a weight to each attribute. Besides, conventional fold-change methods were also applied as controls. The identified genes were further submitted to enrichment analysis for KEGG pathways. The average accuracy of ML models ranges from 86.30% to 91.22%. The overlap ratio of the identified genes between ML and conventional methods ranges from 19.7% to 21.3%. ML exclusively identified oxidative phosphorylation genes in the AD pathway. Our results highlighted the deficiency of oxidative phosphorylation in AD and suggest that ML should be considered as complementary to the conventional fold-change methods in transcriptome studies.

## Introduction

Alzheimer's disease (AD) is a neurodegenerative disease that usually starts gradually around the age of 65 and causes around 70% of dementia cases. Over 20 years, the Aβ amyloid hypothesis dominated the direction of research and drug development in AD. Briefly, APP excision by β- and γ-secretases sequentially yields 40 and 42 amino Aβ monomers, which in turn accumulate into amyloid fibrils and causes downstream tau hyperphosphorylation and neurotoxicity, under the condition of insufficient degradation of Aβ. Although Aβ amyloid and tau hypotheses are still the major focuses of clinical trials^1^, the high failure rate (205 phase 3 trials completed, terminated, withdrawn, and only one approved by FDA up to Feb 2020, http://clinicaltrials.gov) pushed the research community for the reappraisal of the Aβ-centered etiology^2,3^.

According to Gong et al. 2018, the collective effects of multiple genes/insults may lead to the development and onset of AD^2^. Thus, multifactorial diagnosis and personalized treatment were emphasized since different combinations of etiological genes/insults may present in each individual. However, due to insufficient knowledge of AD's full spectrum, there is an urgent need to decipher the mechanism and risk factors of AD.

Machine learning (ML) is the process that computer systems use algorithms and statistical models to perform a prediction relying on patterns and inference without using explicit instructions. The application of ML on AD is focused on the diagnosis of AD from neuroimaging^4^. Despite the fact that the emergence of a wide range of biological data of AD, including genomic profiling and electronic health records, a comprehensive understanding of AD's mechanism from ML is so far unrealized, majorly due to the lack of needed data density^5^. We have previously identified MMP14 and dystonin potentially modulate the crosstalk between diabetes and AD by meta-analysis^6,7^. In this study, we applied ML to a publically available transcriptome dataset from AD postmortem to uncover the complex genetic network and compare the results with conventional fold-change (FC) methods.

## Methods

### Data source

The gene expression profile of the prefrontal cortex brain tissues of 310 AD patients and 157 non-demented control samples were retrieved from the GSE33000 dataset^8^ of the National Center for Biotechnology Information (NCBI) Gene Expression Omnibus (GEO) database. This dataset was selected. The processed data, which have been adjusted for the age, gender, RIN, pH, PMI, batch, and preservation of the samples, were downloaded from the Sample table. This dataset contains 39,279 detected probes, of which 13,798 were annotated, and a total of 9969 genes were profiled, while 31 probes were omitted due to mapping to more than one gene.

Another publically available microarray dataset GSE84422^9^, which profiled PFC from 56 postmortems with varying degrees of AD pathological abnormalities, was utilized as the unseen dataset to verify our models. The samples were classified into control or AD by CDR, Braak, and CERAD. Notably, due to the difference of microarray used, out of the 9966 attribute genes of the training dataset, 3680 genes were not profiled in the testing dataset. To conduct the testing, these 3680 gene profiles were artificially added with FC assigned as "1" for all samples.

### Machine learning

RapidMiner Studio version 9.5 (WIN64 platform) was registered to Jack Cheng and was executed under the Windows 10 operating system with Intel Core i3-3220 CPU and 16 GB RAM. In addition to the samples' age and sex, the 9969 profiled genes were assigned as the regular attributes (potential contributing factors to be analyzed in modeling operator) in the modeling. The disease status (1 = AD; 0 = non-AD CTRL) was assigned as the Label attribute (the predicted class in modeling operator). The sample ID was assigned as the ID attribute (assigning the identity of the sample). The input matrix is supplied as Supplementary File 1.

Seven predictive operators or combinations of RapidMiner Studio operators were used to establish predictive models from the input matrix and assign a weight to each attribute. They were (1) AdaBoost + Decision Tree, (2) AdaBoost + Rule Induction, (3) AdaBoost + Decision Stump, (4) Generalized Linear Model, (5) Logistic Regression, (6) Gradient Boosted Trees, and (7) Random Forest + Weight by Tree Importance. The parameters of these operators are listed in the Parameters sheet of Supplementary File 2. Notably, in the Random Forest model, the number of trees was 500, and the depth of split was set to '-1', which means the maximal depth parameter puts no bound on the depth of the trees. Moreover, the Generalized Linear Model is a regularized GLM, and the elastic net penalty was used for parameter regularization. Other operators under the category Models / Predictive were abandoned in this study due to the reasons listed in the Models sheet of Supplementary File 2.

The model's performance was estimated by cross-validation of models, which contains two subprocesses: a training subprocess and a testing subprocess. The training subprocess produces a trained model to be applied to the testing subprocess for the performance evaluation. In this study, the samples were randomly divided into ten subsets, with an equal number of samples. Each of the ten subsets was iterationaly used in the testing subprocess to evaluate the trained model from the other nine subsets. The convergence of each model's iteration was recorded and summarized in Supplementary File 3, which describes how genes were aggregated from these iterations. The performance of a model can be evaluated by its accuracy, precision, and recall, where accuracy = (TP + TN)/(TP + FP + FN + TN), precision = TP/(TP + FP), recall = TP/(TP + FN), T = true, F = false, P = positive, and N = negative. The setup diagrams of the seven predictive models are illustrated in Supplementary File 4.

### Conventional fold-change method

The fold-change (FC) was defined as the average of gene expression of AD samples relative to that of control samples. Student’s T-test was used to calculate the significance of FC. Non-significant FCs (p > 0.05) were neglected.

### Gene enrichment analysis

The gene list was used as the input to STRING: functional protein association networks^10^ (https://string-db.org/). For the global enrichment analysis, gene symbols with weight/expression levels were submitted to the “Proteins with Values / Ranks” module. For KEGG^11,12^ enrichment analysis, gene symbols were submitted to the “Multiple Proteins by Names / Identifiers” module.

## Results

### Identifying AD-predictive genes by ML

**Figure 1:**
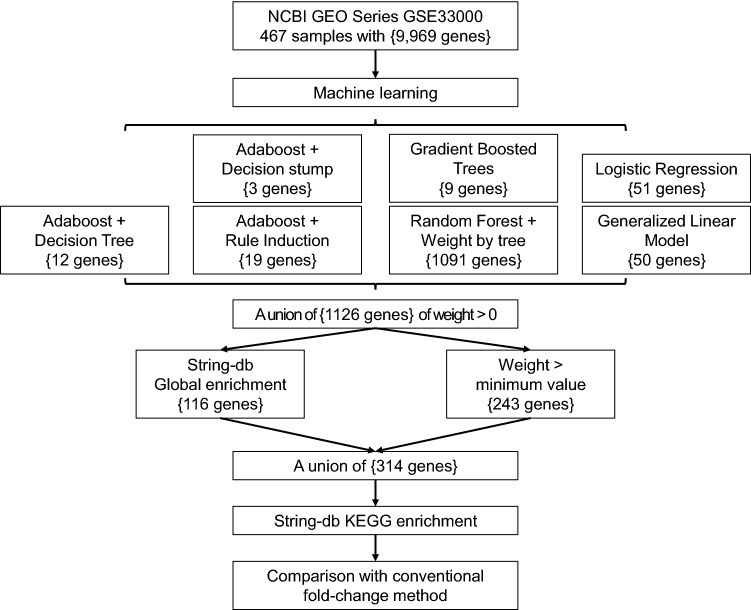
The study design and workflow of identifying AD-predictive genes by ML. The curly brackets indicate the number of genes that passed the criteria or were identified in the ML models.

**Figure 2:**
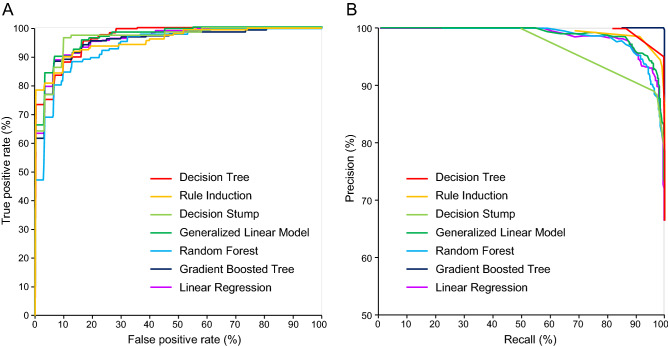
The performance of various ML models. **A)** ROC curves and **B)** precision recall (PR) curves of the ML models used in this study.

We developed a workflow (Fig. 1) to identify AD-predictive genes by ML, and each of the seven predictive operators or combinations of operators produced a gene list along with the weight of predictive contribution. The full lists are provided in the sheets of Generalized Linear Model, Logistic Regression, Rule Induction, Decision Stump, Decision Tree, Gradient Boosted Trees, and Weight of Random Forest of Supplementary File 2. The average accuracy of these models ranges from 86.30% to 91.22%, and the Performance sheet of Supplementary File 2 summarizes the accuracy, precision, and recall of each model, while ROC curves and precision recall curves are shown in Fig. 2. Combing the genes from the seven models, we got a union of 1126 non-redundant genes with weight > 0 (the Non-redundant Genes sheet of Supplementary File 2). To further extract the more representative genes, those genes satisfying both conditions, 1) genes with the weight of the minimum value (i.e., 0.001), and 2) genes without a presence in the global enrichment analysis (the Global Enrichment sheet of Supplementary File 2), were filtered out. Finally, we reached a list of 314 genes (the Genes sheet of Supplementary File 5).

We conducted the analysis of variance (ANOVA) test to determine the probability for the null hypothesis of the equal performance of the different ML models. The ANOVA result (f = 1.558, prob = 0.174, alpha = 0.050) could not reject the null hypothesis, indicating that the difference between the performance of the different ML models is not significant. The process was exported as “ANOVA.rmp” and was uploaded to GitHub at https://github.com/JackCheng-TW/RapidMiner-files/Process/.

To check if our findings are not unique to a single dataset, we took another microarray-profiled PFC dataset GSE84422 as an unseen dataset to verify our models. Although nearly one-third of the training genes are missing in the test dataset, GSE84422 is currently the 2nd largest one after GSE33000. Upon testing, the accuracy was 28.57%, 58.93%, 76.79%, 82.14%, 71.43%, 44.64%, and 69.64% for decision tree, random forest, gradient boosted tree, generalized linear model, linear regression, decision stump, and rule induction, respectively. Since there are only a few attributes in decision tree/stump, missing one or two may largely limit the model performance. In contrast, models with more attributes like GLM outperform the others. The results indicate some models' generalizability and the difficulty of applying ML models on cross-platform datasets. The testing dataset "GSE84422_testing.xls" and exported processes were uploaded to GitHub at https://github.com/JackCheng-TW/RapidMiner-files/testing/.

### ML compensates conventional FC methods in gene identification

**Figure 3:**
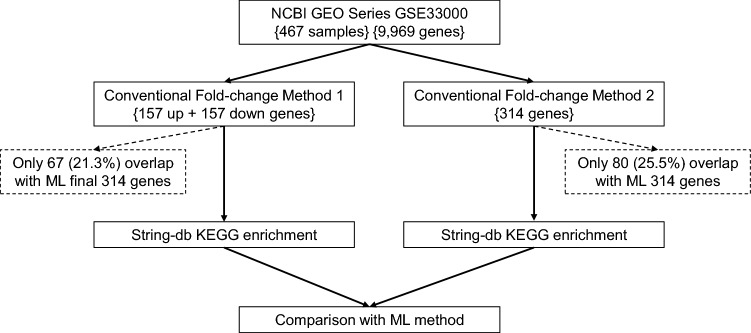
The workflow of identifying dysregulated genes by the conventional FC method. The curly brackets indicate the number of genes that passed the criteria or were identified in each step. The overlap of identified genes between ML and the FC method is shown in dashed squares.

To compare the differences in gene identification between ML and conventional FC-based methods, we adopted two independent strategies, as illustrated in Fig. 3. In one way, the uppermost 157 genes and the bottommost 157 genes of fold change were selected (Genes sheet of Supplementary File 6). In the other way, we selected 314 DEGs by firstly filtering with the fold change cutoffs 1.2, and followed by the rank of the p values (Genes sheet of Supplementary File 7). Surprisingly, there were only 67 (21.3%) or 80 (25.5%) genes overlapped with the ML-derived 314 genes for the two conventional FC-based methods, respectively.

Next, to figure out the differences in enriched pathways, the final gene list from ML and those from the two conventional FC-based methods were submitted to KEGG pathway enrichment analysis, respectively. The top 15 enriched KEGG pathways are summarized in Table 1, while the KEGG sheets in Supplementary Files 5, 6, 7 provide the full results. As anticipated, most of the pathways enriched by ML-derived genes are not redundant to conventional FC-based methods. Interestingly, the KEGG Alzheimer's pathway (hsa05010) was only enriched by the ML-derived genes, not conventional methods. However, this does not imply that ML is superior to or can replace the conventional methods since the latter also exclusively enriched several AD-related pathways, such as complement cascades (hsa04610), cytokine-cytokine receptor interaction (hsa04060), and phagosome (hsa04145). The mutual exclusivity of critical pathways demonstrates that ML compensates the conventional FC methods in gene identification.

**Table 1:** Enriched KEGG pathways of genes identified by machine learning and conventional fold-change (FC) methods, respectively.

	Machine learning	FC method 1	FC method 2
1	Alzheimer's disease	Complement and coagulation cascades	GABAergic synapse
2	Parkinson's disease	Staphylococcus aureus infection	Morphine addiction
3	Huntington's disease	Phagosome	MAPK signaling pathway
4	Thermogenesis	Pertussis	Retrograde endocannabinoid signaling
5	Oxidative phosphorylation	Legionellosis	Nicotine addiction
6	Neurotrophin signaling pathway	Rheumatoid arthritis	Butanoate metabolism
7	MAPK signaling pathway	Malaria	
8	Acute myeloid leukemia	Systemic lupus erythematosus	
9	Non-alcoholic fatty liver disease (NAFLD)	Prion diseases	
10	Retrograde endocannabinoid signaling	Cytokine-cytokine receptor interaction	
11	FoxO signaling pathway	TNF signaling pathway	
12	Endometrial cancer	Kaposi's sarcoma-associated herpesvirus infection	
13	Alcoholism	MAPK signaling pathway	
14	Influenza A	Ras signaling pathway	
15	Serotonergic synapse	Influenza A	

### ML highlights oxidative phosphorylation genes in the AD pathway

**Figure 4:**
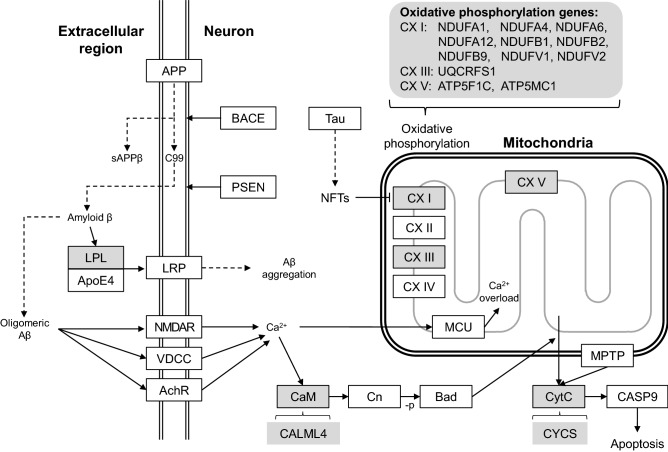
The ML-identified genes enrich the KEGG AD pathway. Proteins or protein complexes are shown in squares. Only a part of the KEGG AD pathway is shown. Dash line arrows indicate transformation, while solid line arrows indicate a reaction. The enriched proteins or protein complexes are drawn in grey, with the ML-identified genes noted at the open bracket near it.

When we looked into ML-derived genes, which enriched the pathways, we found a considerable overlap of genes between the ML-exclusive pathways (Table 2). These genes are ATP5C1, ATP5G1, NDUFA1, NDUFA4, NDUFA6, NDUFA12, NDUFB1, NDUFB2, NDUFB9, NDUFV1, NDUFV2, and UQCRFS1. They belong to the oxidative phosphorylation pathway (hsa00190), which is also a part of the KEGG Alzheimer’s pathway (Fig. 4). Among them, NDUFA1, NDUFA4, NDUFA6, NDUFA12, NDUFB1, NDUFB2, NDUFB9, NDUFV1, NDUFV2 belong to the OXPHOS protein complexes (CX) I of the electron transport chain (ETC); while UQCRFS1 belongs to CX III of ETC. Moreover, ATP5C1 and ATP5G1 belong to the ATP synthase (CX V).

**Table 2:** Overlapping of ML-identified genes in the enriched KEGG pathways. “O” denotes the presence of the gene.

KEGG gene	Alzheimer's disease	Thermogenesis	Oxidative phosphorylation	Non-alcoholic fatty liver disease (NAFLD)
ATP5C1	O	O	O	
ATP5G1	O	O	O	
CALML4	O			
CYCS	O			O
LPL	O			
NDUFA1	O	O	O	O
NDUFA12	O	O	O	O
NDUFA4	O	O	O	O
NDUFA6	O	O	O	O
NDUFB1	O	O	O	O
NDUFB2	O	O	O	O
NDUFB9	O	O	O	O
NDUFV1	O	O	O	O
NDUFV2	O	O	O	O
UQCRFS1	O	O	O	O
ACTB		O		
RPS6KA1		O		
SMARCA4		O		
SOS1		O

### Co-predictive partners of the CX genes

**Figure 5:**
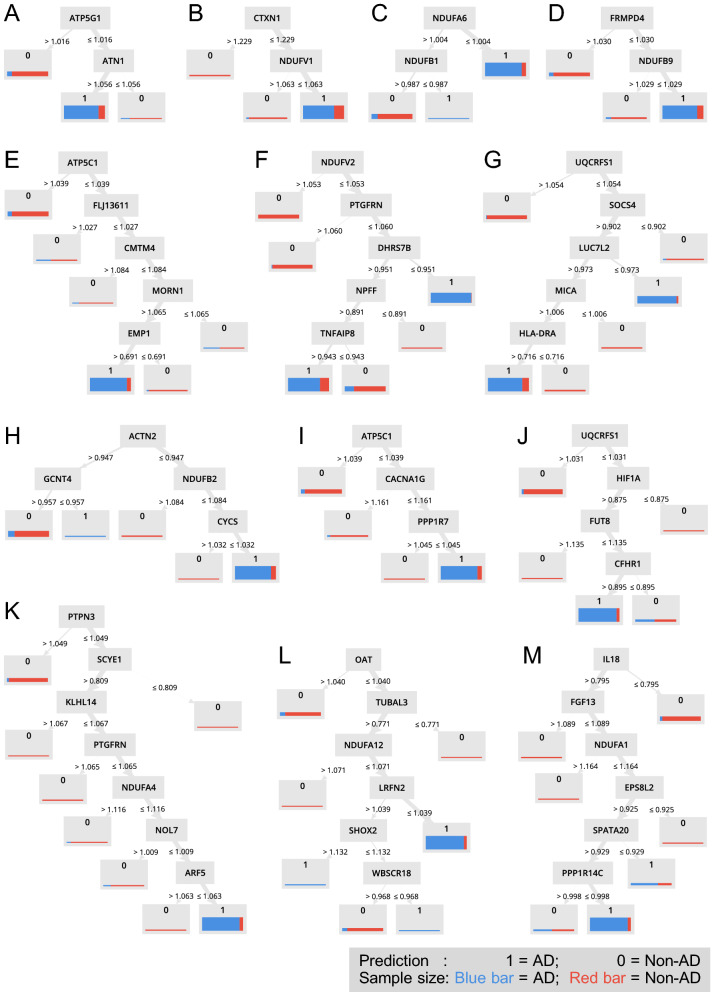
Slightly down-regulated oxidative phosphorylation genes predict AD. (**A**) to (**M**) The decision trees from the random forest model containing NDUFA1, NDUFA4, NDUFA6, NDUFA12, NDUFB1, NDUFB2, NDUFB9, NDUFV1, NDUFV2, UQCRFS1, ATP5F1C, and ATP5MC1. The prediction outcome is denoted by 0 and 1, where 1 = AD, and 0 = Non-AD. The sample size is denoted by the thickness of the bar, while the sample type is denoted by blue or red, where blue bar = AD, and red bar = Non-AD.

Random Forest produces decision trees, which use combinations of the Attribute value, i.e., expression of genes, to predict the sample Label, i.e., AD or not. Figure 5 shows 13 decision trees involving ETC complexes subunit genes, and Table 3 summarizes the 12 CX genes and other 37 predictive genes in these trees. Notably, 32 out of the 37 genes are relevant to AD. The AD-relevance is established by association studies of the expression, genomics, or metabolomics, respectively, with references listed in Table 3.

**Table 3:** Oxidative phosphorylation genes and their companions identified in machine learning.

Gene	Full name	AD	Association			Refs.
			E	G	M	
ATP5F1C*	ATP synthase subunit gamma, mitochondrial	O				
ATP5MC1*	ATP synthase F(0) complex subunit C1, mitochondrial	O				
NDUFA1	NADH dehydrogenase [ubiquinone] 1 alpha subcomplex subunit 1	O				
NDUFA12	NADH dehydrogenase [ubiquinone] 1 alpha subcomplex subunit 12	O				
NDUFA4	Cytochrome c oxidase subunit NDUFA4	O				
NDUFA6	NADH dehydrogenase [ubiquinone] 1 alpha subcomplex subunit 6	O				
NDUFB1	NADH dehydrogenase [ubiquinone] 1 beta subcomplex subunit 1	O				
NDUFB2	NADH dehydrogenase [ubiquinone] 1 beta subcomplex subunit 2, mitochondrial	O				
NDUFB9	NADH dehydrogenase [ubiquinone] 1 beta subcomplex subunit 9	O				
NDUFV1	NADH dehydrogenase [ubiquinone] flavoprotein 1, mitochondrial	O				
NDUFV2	NADH dehydrogenase [ubiquinone] flavoprotein 2, mitochondrial	O				
UQCRFS1	Cytochrome b-c1 complex subunit Rieske, mitochondrial	O				
ACTN2	Alpha-actinin-2		O	O		^64,65^
ARF5	ADP-ribosylation factor 5		O			^66^
ATN1	Atrophin-1		O			^14^
CACNA1G	Voltage-dependent T-type calcium channel subunit alpha-1G		O			^67^
CFHR1	Complement factor H-related protein 1		O			^68^
CMTM4	CKLF like MARVEL transmembrane domain containing 4		O			^69^
CTXN1	Cortexin-1		O			^16^
DHRS7B	Dehydrogenase/reductase SDR family member 7B		O			^70^
EMP1	Epithelial membrane protein 1			O		^71^
EPS8L2	Epidermal growth factor receptor kinase substrate 8-like protein 2		O			^72^
FGF13	Fibroblast growth factor 13		O			^67^
FLJ13611*	Trafficking protein particle complex 13			O		^73^
FRMPD4	FERM and PDZ domain-containing protein 4		O			^18^
FUT8	Alpha-(1,6)-fucosyltransferase		O			^74^
GCNT4	Beta-1,3-galactosyl-O-glycosyl-glycoprotein beta-1,6-N-acetylglucosaminyltransferase 4					
HIF1A	Hypoxia-inducible factor 1-alpha		O	O		^75,76^
HLA-DRA	HLA class II histocompatibility antigen, DR alpha chain		O	O		^67,77^
IL18	Interleukin-18		O			^78^
KLHL14	Kelch-like protein 14					
LRFN2	Leucine-rich repeat and fibronectin type-III domain-containing protein 2			O		^79^
LUC7L2	Putative RNA-binding protein Luc7-like 2		O			^80^
MICA	MHC class I polypeptide-related sequence A					
MORN1	MORN repeat containing 1		O			^81^
NOL7	Nucleolar protein 7		O			^82^
NPFF	Pro-FMRFamide-related neuropeptide FF			O		^83^
OAT	Ornithine aminotransferase, mitochondrial				O	^45^
PPP1R14C	Protein phosphatase 1 regulatory subunit 14C			O		^84^
PPP1R7	Protein phosphatase 1 regulatory subunit 7		O			^85^
PTGFRN	Prostaglandin F2 receptor negative regulator		O			^86^
PTPN3	Tyrosine-protein phosphatase non-receptor type 3		O			^18^
SCYE1*	Aminoacyl tRNA synthase complex-interacting multifunctional protein 1					
SHOX2	Short stature homeobox protein 2					
SOCS4	Suppressor of cytokine signaling 4		O			^87^
SPATA20	Spermatogenesis-associated protein 20					
TNFAIP8	Tumor necrosis factor alpha-induced protein 8			O		^88^
TUBAL3	Tubulin alpha chain-like 3	O				
WBSCR18*	DNAJC30 interacts with ATP synthase and links mitochondria to brain development		O			^89^

An “O” denotes the present knowledge that supports the involvement or association of the gene with Alzheimer’s disease. The category item “AD” indicates the involvement of the gene in the KEGG AD pathway, while “E”, “G”, and “M” indicate the evidence of association studies of the expression, genomics, and metabolomics. An * sign indicates the presence of another preferred name in the String-db. The alternative names are shown in the parentheses. ATP5F1C (ATP5C1); ATP5MC1 (ATP5G1); FLJ13611 (TRAPPC13); SCYE1 (AIMP1); WBSCR18 (DNAJC30).

Figure 5A shows that the expression of ATP5G1 and ATN1 predicts AD. Although the exact function of ATN1 is unknown, it may act as a transcriptional co-repressor in neurons^13^. Moreover, alternative splicing of ATN1 was significantly detected in the frontal lobe of AD postmortem^14^. Figure 5B shows that the expression of NDUFV and CTXN1 predicts AD. CTXN1 encodes cortexin-1 and may mediate signaling of cortical neurons during forebrain development^15^, and it is highly dysregulated in the aging brain^16^. Figure 5C shows that slightly downregulation of two CX I genes, NDUFA6 and NDUFB1 predicts AD. Figure 5D shows that the expression of NDUFB9 and FRMPD4 predicts AD. FRMPD4 positively regulates dendritic spine morphogenesis and involves in excitatory synaptic transmission^17^. Besides, the expression of FRMPD4 was found to be significantly altered in the AD hippocampus^18^. Other AD-predictive genes in these decision trees will be discussed in groups according to their biological functions.

## Discussion

We conducted machine learning (ML) analyses to train AD case/control classifiers using transcriptomic data and then compared the ML-derived gene features with that from the conventional differential expression analysis. ML exclusively highlighted oxidative phosphorylation but could not fully include the findings from the conventional methods. The pathways involving the identified genes and the limitation of the study are discussed below.

### Oxidative phosphorylation

Oxidative phosphorylation in eukaryotes takes place at the electron transport chain in the mitochondrion. The oxidation of NADH or succinate from the citric acid cycle is the energy source of ATP synthase. During this process, several mitochondrial inner-membrane-embedded complexes, including CX I and CX III, pump protons out from the inner membrane to establish proton gradient, while CX V utilizes the energy of the influx of protons to generate ATP from ADP^19^.

Abnormal mitochondrial morphology and functions, including glucose metabolism and ROS production, have been identified as early hallmarks of AD^20,21^. These phenotypes are directly related to the disruption of glycolytic processes and the impairment of the ETC complexes. In the '90 s, most research efforts have been devoted to investigating the role of CX IV in AD^22,23^. However, the evidence is not conclusive on whether dysregulation of any single ETC complex dominates AD progress. For example, besides expression, several mutations in ETC complex subunit genes may impair the complex activity^24^. Moreover, the ETC complex's dysregulation seems to be brain-region dependent, e.g., CX IV has no significant decrease in the temporal lobe, and CX I–III are decreased at certain cortex locations of AD^24^.

Recent studies also highlighted CX I's role, especially its deregulation, is tau-dependent in contrast to the Aβ-dependent CX IV^25^. Moreover, an SNP association study demonstrated the AD association for complex I genes but not for complexes II–V^26^. Furthermore, from a postmortem study of 18 AD and 44 controls, the downregulation of CX I-V in the hippocampus was identified^27^. However, the expression of CX I genes may not be monotonic during the AD progression. From a postmortem study of twelve AD and six controls, CX I genes are reported to decrease in the early stage and increase in the frontal cortex of definite AD patients^28^.

When we only see the symptom, most things look complex, especially the case for ETC complexes in AD. Could ML guide us through this misty forest with the aid of the Random Forest model by finding out potential partners of CX genes in predicting AD?

### Neural maintenance or transmission

Among the AD-predictive genes, CACNA1G, FGF13, LRFN2, NPFF, and SHOX2 participate in neural maintenance or transmission. CACNA1G encodes voltage-dependent T-type calcium channel subunit alpha-1G. FGF13 is a fibroblast growth factor and plays a critical role in neuron polarization and migration^29^. LRFN2 promotes neurite outgrowth and increases the expression of the NMDA receptor^30^. NPFF is a neuropeptide, while SHOX2 may be a growth regulator in the neural system and involves processing somatosensory information^31^. In AD, pathological hallmarks include synaptic failure and neuronal loss^32^. Moreover, the critical role of mitochondria in supporting synaptic, as well as the evidence of dysfunction of mitochondria from both clinical postmortem^33^ and animal models^34^ of AD, support the mitochondria-synapse hypothesis of AD. Our findings that simultaneous dysregulation of CX and neuronal genes predict AD supports this hypothesis.

### Immune system

The innate immunity, especially neuroinflammation mediated by microglia, is considered a hallmark of AD, whereas the role of the adapted immunity in AD is not conclusive^35^. Among the AD-predictive genes, CFHR1, CMTM4, HLA-DRA, IL18, MICA, MORN1, SCYE1, and SOCS4 participate in immunity. CMTM4 regulates PD-L1 protein^36^, which binds to PD-1 and suppresses the T-cells' adaptive arm, while HLA-DRA presents the extracellular-protein-derived peptides to, and MICA presents the stress-induced self-antigen to T-cells, respectively^37^. Moreover, MORN1 modulates functional Ca^2+^ influx in T cells upon activation of T-cell receptors^38^. IL18 and SCYE1 (AIMP1) are pro-inflammatory cytokines, while SOCS4 is part of a negative feedback system that regulates cytokine signal transduction^39^. CFHR1 is an inhibitor of the complement pathway that blocks C5 convertase and controls complement activation along with complement factor H^40^. Our results indicate that the dysregulation of both innate and adaptive immunity genes may cooperate with CX genes to advance AD progression.

### Phosphatase regulators

In AD, hyperphosphorylation of the microtubule-associated proteins, especially tau, disrupts the microtubules' assembly in neurons. Moreover, significantly lower type 1 phosphatase (PP1) activity in AD brains suggests the critical role of dysfunctional phosphatases in AD^41^. Among the AD-predictive genes, PPP1R14C and PPP1R7 belong to PP1 regulatory subunit 14 and subunit 7, respectively. Our results indicate that the dysregulation of PPP1R14C and PPP1R7, along with CX genes, may further advance AD progression by aggravating the microtubule-associated proteins' hyperphosphorylation.

### Protein glycosylation

Protein glycosylation is a ubiquitous posttranslational modification of site-specific attachment of glycans and regulates the protein's folding and function. During the protein transport from Endoplasmic Reticulum to the Golgi apparatus, a series of attachment of oligosaccharides maturates a wide variety of complex N- or O-glycans. An N-glycosylation denotes the glycan's attachment to the amide nitrogen of an asparagine residue of the protein, whereas an O-glycosylation denotes the attachment to the oxygen atom of serine or threonine residues. Abnormal N- and O-glycosylation has been reported in AD^42,43^. Among the AD-predictive genes, FUT8 and GCNT4 mediate glycosylation in the Golgi apparatus. FUT8 catalyzes the addition of fucose to the GlcNAc residue, while GCNT4 is a glycosyltransferase mediating O-glycan branching^44^. Thus, the dysregulation of FUT8 and GCNT4 may aggravate AD progression by abnormal glycosylation under the condition of CX deficiency.

### Other mitochondria machinery

Notably, among the AD-predictive genes, there are two mitochondrial genes besides the CX: Ornithine aminotransferase (OAT) and DnaJ homolog subfamily C member 30 (DNAJC30/ WBSCR18). OAT converts ornithine into pyrroline-5-carboxylate (P5C), which can serve as the precursor of proline and glutamate. Furthermore, since ornithine is an intermediate product in the urea cycle, OAT dysregulation may lead to abnormalities of both energy production machinery and the supply of neural transmitters. Recently, the OAT substrate ornithine has been proposed as an early diagnostic biomarker of AD^45^, and altered expression of the urea cycle enzymes have been identified in sporadic AD brains^46^. Our finding that simultaneous downregulation of OAT and CX I predicts AD indicates that the deficiency of the urea cycle and CX may co-operate to advance AD.

Meanwhile, DNAJC30 has been recently identified as an auxiliary component of ATP-synthase machinery in the mitochondria^47^. The removal of Dnajc30 in mice resulted in hypofunctional mitochondria, decreased integrity of CXs, and abnormal neocortical pyramidal neurons^47^. Our finding that the simultaneous downregulation of DNAJC30 and CX I predicts AD also supports the mitochondria deficiency hypothesis of AD.

### Slightly dysregulated CX genes predict AD

From an overall observation on the CX-related decision trees (Fig. 5), a combination of down-regulated CX components and one or several partner genes mentioned above predicts AD. Notably, the margin is not conventional twofold, 1.5-fold, or even 1.2-fold. The margin is very subtle, and this is why the conventional FC method cannot identify them. With the criteria of p < 0.05 and FC 1.2, 1.5, or 2, the numbers of DEGs of the model dataset GSE33000 are 418, 10, and 0, respectively, as shown in Supplementary File 10. We compared the 418 DEGs with the ML 314 genes in Supplementary File 5 and found the number of intersection genes to be 60 (19.1%), which was compatible with the results of the conventional method 1 (21.3%) and the conventional method 2 (25.5%). Furthermore, it is difficult to identify complicated rules by conventional methods. Therefore, we suggest adopting machine-learning algorithms, especially decision trees, rule induction, and random forest, as complementary methods in transcriptome studies.

### Limitations

There are several limitations to the interpretation of the results. (1) The samples are primarily of Caucasian ancestry. The biased sample race may limit the results to be applied to other races. (2) The samples are from the postmortem of a specific brain region. Since expressional heterogeneity, this may limit the results to be applied to other brain regions. (3) Due to the same reason, the results can hardly be applied to patient diagnosis purposes. (4) For the future application of the study pipeline, at least hundreds of samples might be required due to ML's nature. (5) ML models predict the patient disease labels but not the involvement of genes in disease, and additional genetic evidence is required to delineate any possible causal/reactive roles of these gene features in AD. (6) The performance difference in the independent dataset could be attributed to the detectable genes of different chip systems and the within-dataset variations. The absence of 36.9% attributes (genes) in the test set largely limited the performance of some models. Moreover, the limitation may also come from the difference in the sampling quality, which is reflected by the within-dataset variation (the average STD/INT were 8.2% and 21% for the modeling set and test set, respectively).

Rapidminer models have also been used to identify the transcriptomic bio-signature of an infectious disease condition in the mammary gland of the cow^48^, with the performance ranging from 53 to 87%, which is compatible with the performance of this study. The differences in strategy majorly lay in whether pre-screening attributes (the so-called feature selection) before applying ML^49^. The benefits of feature selection include simplifying models, shorter training times, and avoidance of high dimensionality problems; however, the feature selection step using the entire dataset may strongly bias all downstream prediction, even when cross-validation is used^50^. Therefore, in this study, we decided to skip the feature selection step to achieve an unbiased understanding of AD.

Since decision trees were the final models to identify potential novel genes in this study, whether the data size is big enough is crucial. According to Vabalas et al.^51^, we conducted a series of train/test split to validate whether arbitrary partial subsets of data could generate decision trees to predict the “unseen” counterpart, with the same parameters used in this study. As shown in Supplementary File 8, the recall rates were saturated at n = 94, i.e., 20% of the total samples, which may imply the sample size was sufficient to conduct this study.

Although we did not combine datasets in this study, appropriate methods used for reducing the batch effect and differences between experiments^52^ should be applied when combing datasets in future studies. We also noticed that random forest analysis dominated the identified gene features, indicating that future similar studies might focus on random forest first. However, other models may supply other 10% genetic cues on the investigator’s demand.

### Hypotheses developed from ML models

To discover and characterize the underlying pathophysiological pathways of AD are the main objectives of genetic research, including this ML study. Based on our findings, we postulate that two novel players, i.e., RNF157 and KIAA1715, may independently participate in AD pathophysiology by mediating the mitogen-activated protein kinase (MAPK) signaling pathway. MAPKs are serine/threonine protein kinases regulating cellular processes in response to environmental stimuli and participate in hallmark events of AD, including tau phosphorylation, Aβ deposition, and chronic inflammation^53,54^.

In the #2 model of RF (Supplementary File 2), RNF157, EPHA2, and hCG_1776018 (also known as PIRT, an uncharacterized phosphoinositide-interacting protein) co-predict AD. EPHA2 is a membrane receptor tyrosine kinase, which regulates migration, adhesion, and blood–brain barrier through MAPK signaling^55^. RNF157 is an E3 ubiquitin ligase that acts as a downstream effector of PI3K/MAPK signaling^56^ and regulates the survival of neurons by ubiquitinating APBB1^57^. Presently, there is no knowledge about the roles of these three genes in AD. We hypothesize that RNF157 may act as the downstream of EPHA2 and hCG_1776018, and regulate neural death upon cellular stress in the AD microenvironment. We also postulate that RNF157 agonist may act as a symptomatic treatment in AD.

In the #230 model of RF (Supplementary File 2), KIAA1715 and MAP3K9 co-predict AD. MAP3K9 is a serine/threonine kinase that is activated by environmental stress and acts as an upstream activator of the MKK/JNK signal transduction cascade regulating apoptosis^58^. MAP3K9 dysregulation has been proposed as a possible marker in AD^59^. KIAA1715 (also known as LNPK) is an endoplasmic reticulum (ER) membrane protein, which stabilizes ER curvature and ER tubular junction network^60,61^. Mutations in KIAA1715 cause neurodevelopmental syndromes, such as intellectual disability and epilepsy^61^. Notably, disruption of ER-mitochondria contact has recently been found in AD postmortem^62^, while restoring ER-mitochondria contact rescues AD animal model^63^. However, there is no knowledge about the role of KIAA1715 in AD. We hypothesize that under the pro-inflammatory microenvironment of AD, KIAA1715 deficiency may lead to instability of ER structure, leading to disruption of ER-mitochondria contact and eventually aggravate AD progression.

## Conclusion

Our study using machine learning techniques on the gene expression profile of the postmortem of the prefrontal cortex brain tissues of AD and controls highlighted the oxidative phosphorylation genes in the AD pathway. These genes were exclusively identified in ML but not in the conventional counterpart. Our results imply that ML should be considered complementary to the conventional FC methods in transcriptome studies. More importantly, we show that hypotheses underlying pathophysiological pathways of AD could be developed by further looking into ML models.

## Supplementary Information

Supplementary Information 1.

Supplementary Information 2.

Supplementary Information 3.

Supplementary Information 4.

Supplementary Information 5.

Supplementary Information 6.

Supplementary Information 7.

Supplementary Information 8.

Supplementary Information 9.

Supplementary Information 10.

Supplementary Information 11.

## Data Availability

All data in this study are included in the supplementary data. The raw data used for machine learning and traditional expression analysis in the CSV format was uploaded as Supplementary File 1. Besides, it is also available from https://github.com/JackCheng-TW/RawData. The independent dataset was uploaded as Supplementary File 9.

## Abbreviations

AD: Alzheimer's disease
ML: Machine learning
OXPHOS: Oxidative phosphorylation
CX: OXPHOS protein complex
FC: Fold-change

## Code availability

The machine learning platform RapidMiner Studio is available at https://rapidminer.com/. The process files in Rapidminer format (.rmp) of this study and the generated models were uploaded to GitHub at https://github.com/JackCheng-TW/RapidMiner-files.
