# Supplementary material for: Machine learning compensates fold-change method and highlights oxidative phosphorylation in the brain transcriptome of Alzheimer’s disease
Source: Sci Rep. 2021 Jul 1;11:13704. doi: 10.1038/s41598-021-93085-z (PMC8249453; doi:10.1038/s41598-021-93085-z)

1) AdaBoost + Decision Tree

Supplementary File 4.

Top Level

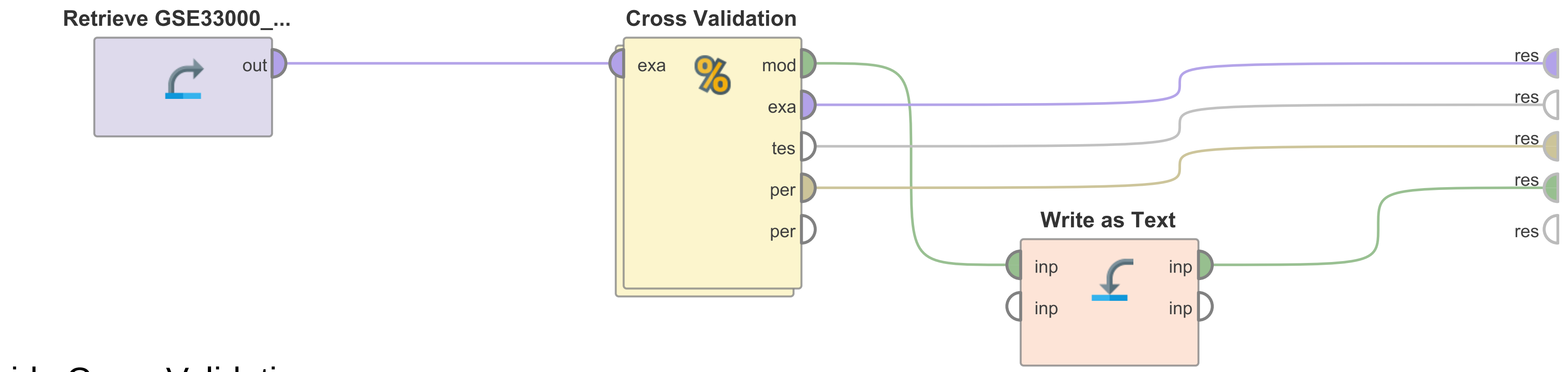

Inside Cross Validation

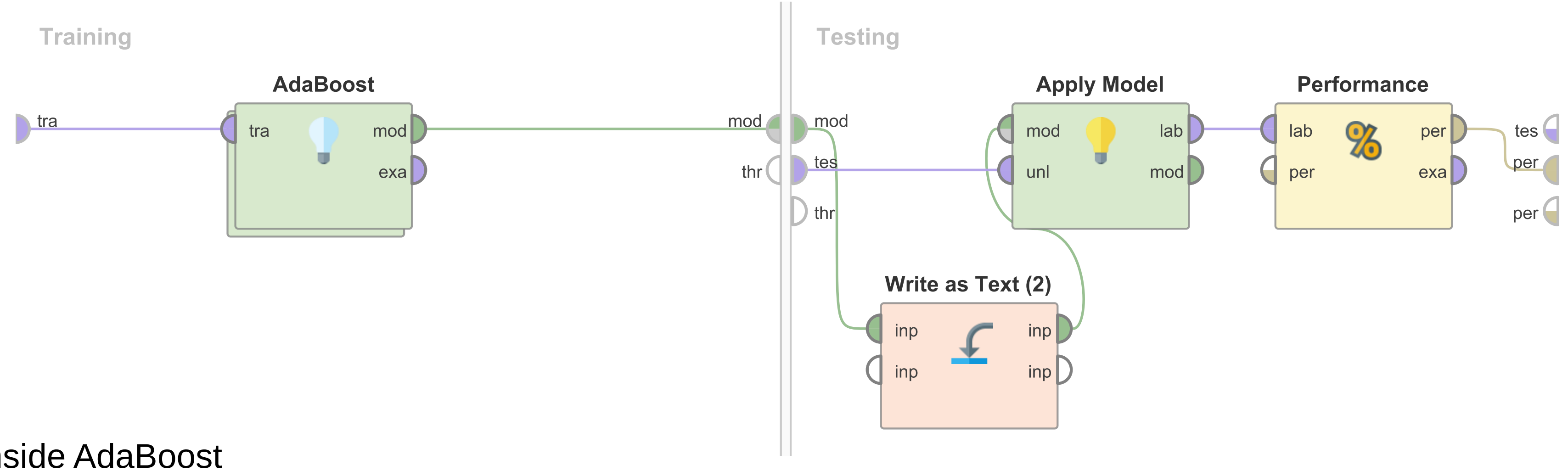

Inside AdaBoost

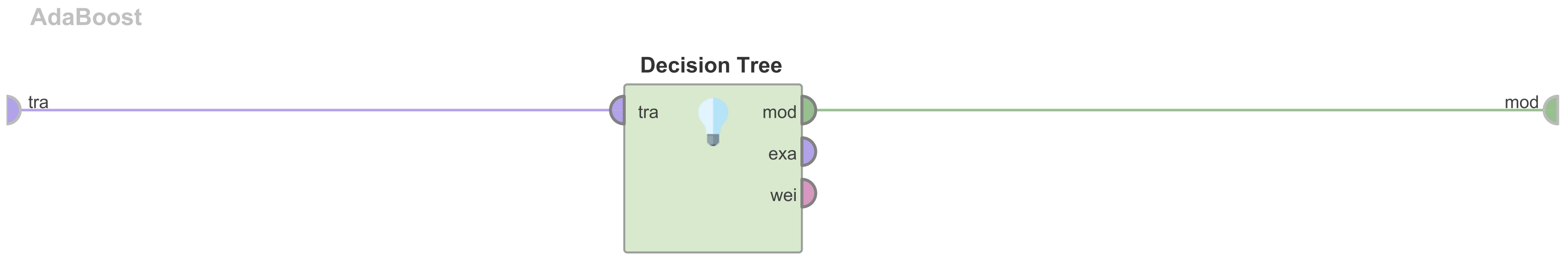

2) AdaBoost + Rule Induction

Top Level

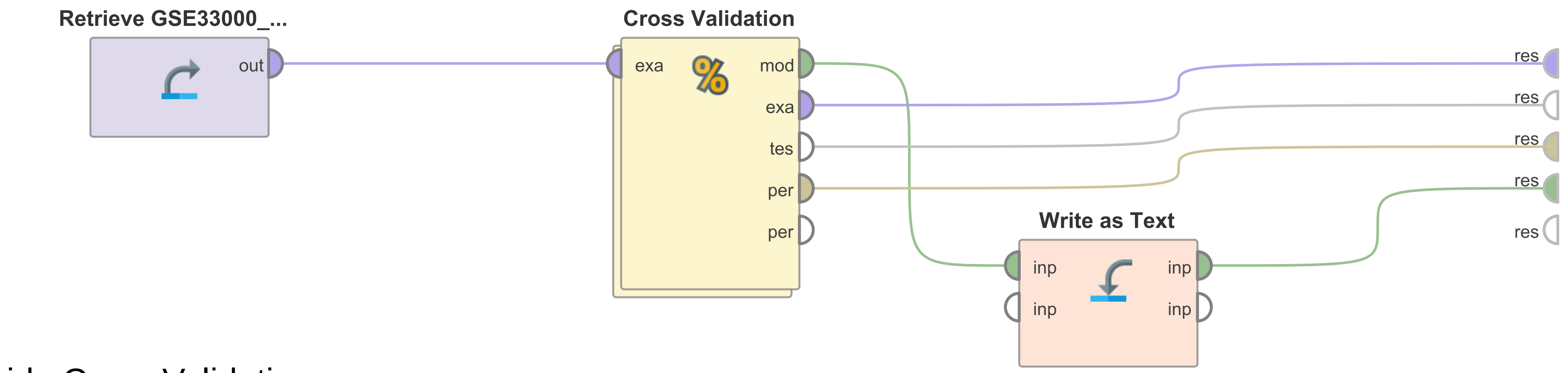

Inside Cross Validation

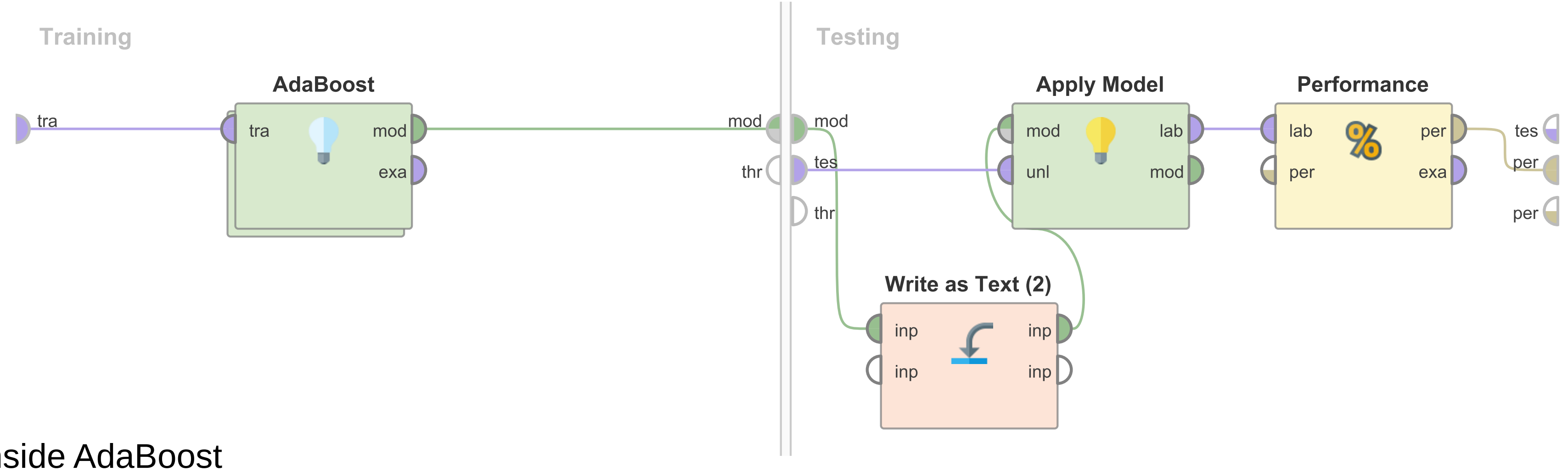

Inside AdaBoost

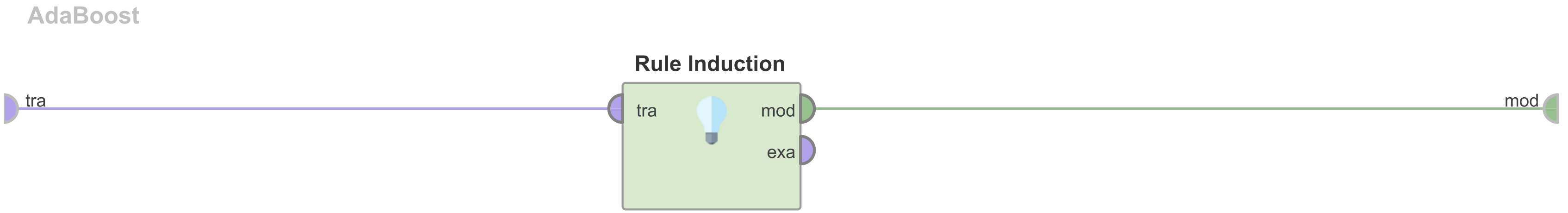

3) AdaBoost + Decision Stump

Top Level

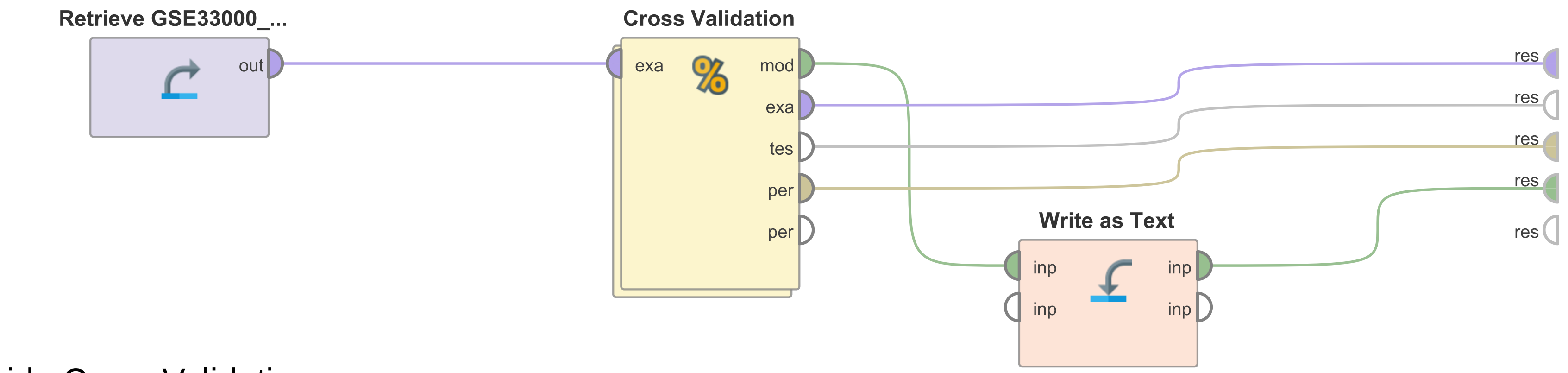

Inside Cross Validation

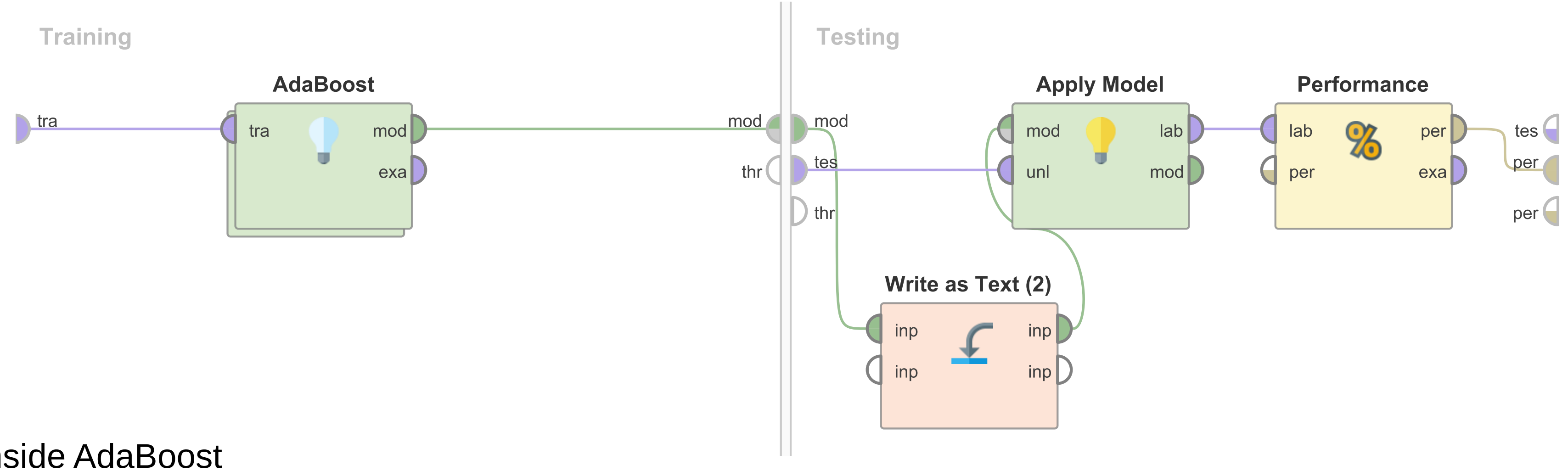

Inside AdaBoost

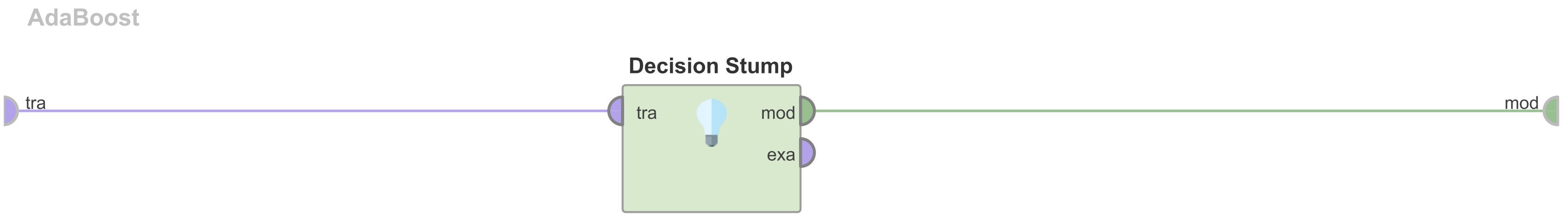

4) Generalized Linear Model

Top Level

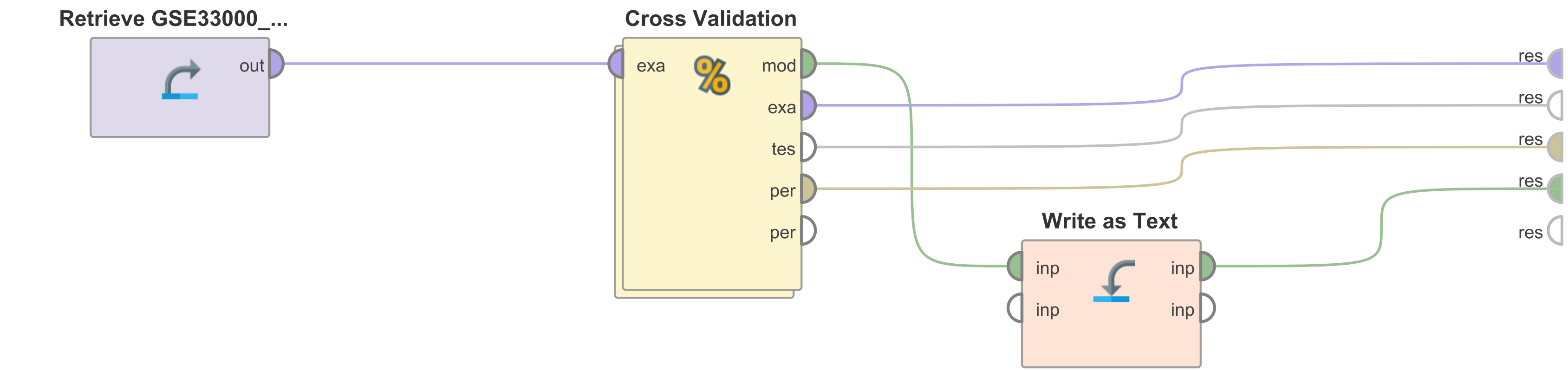

Inside Cross Validation

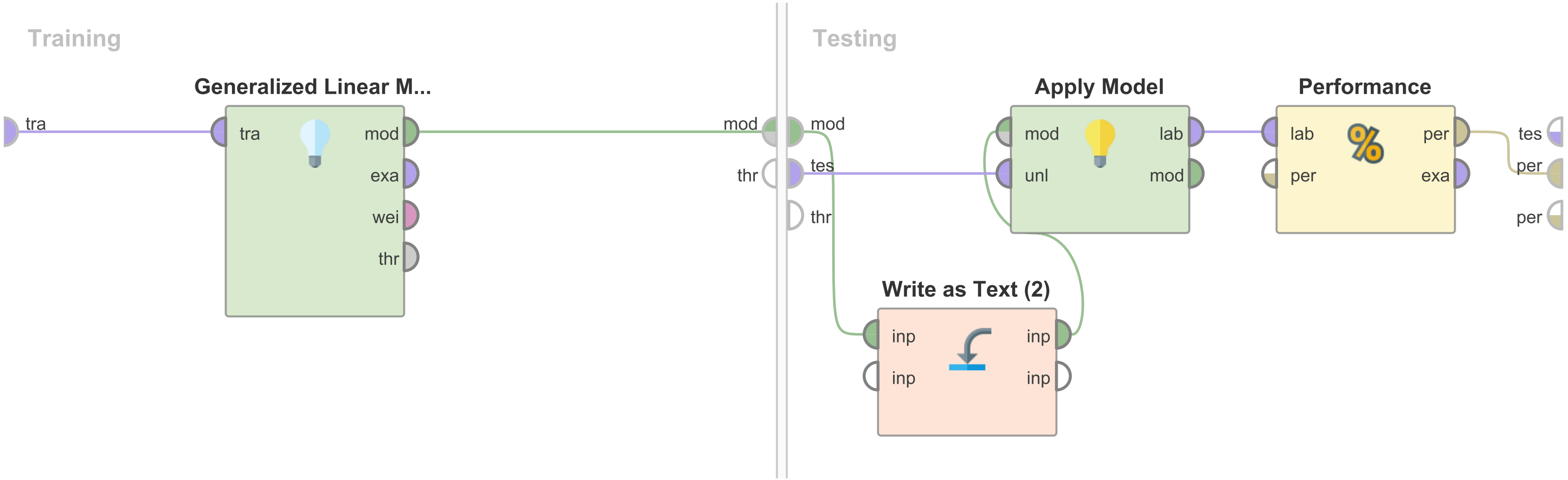

5) Logistic Regression

Top Level

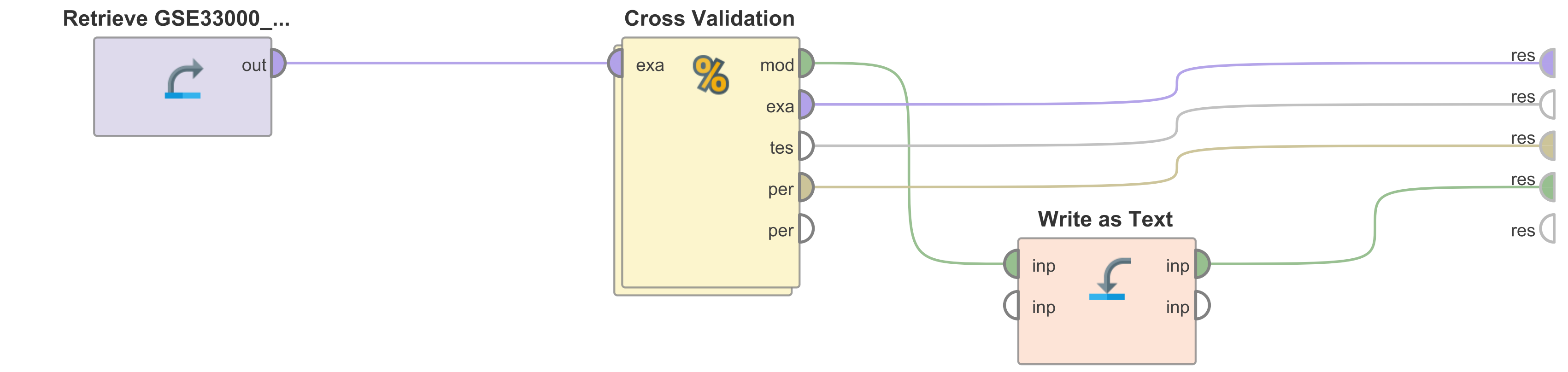

Inside Cross Validation

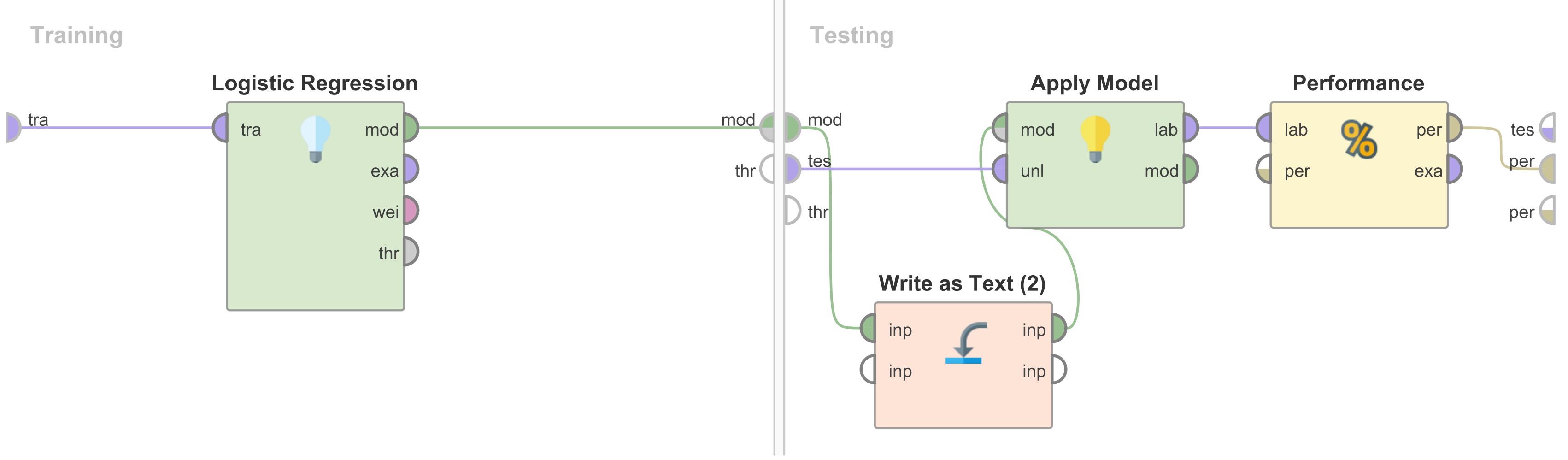

6) Gradient Boosted Trees

Top Level

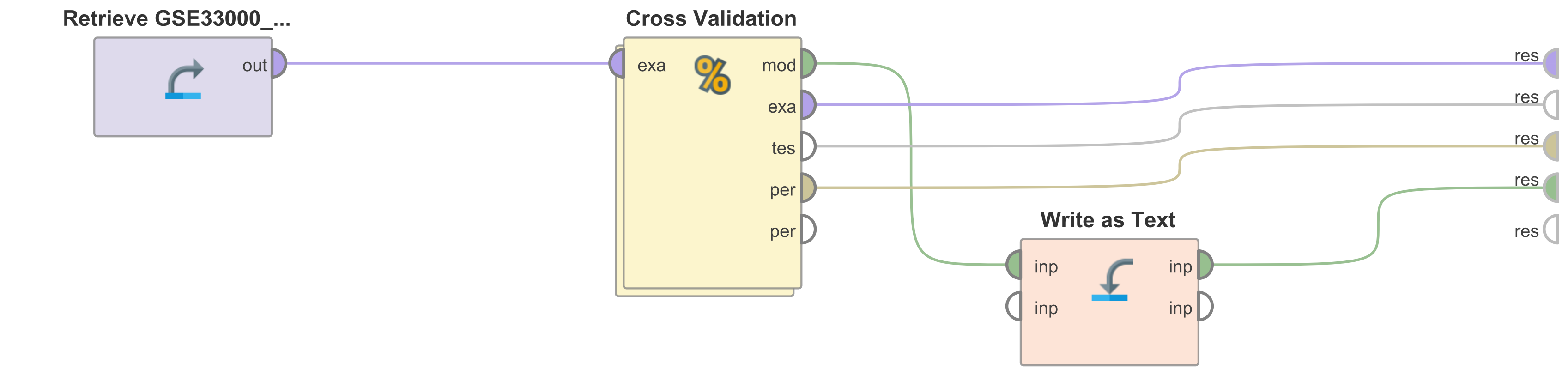

Inside Cross Validation

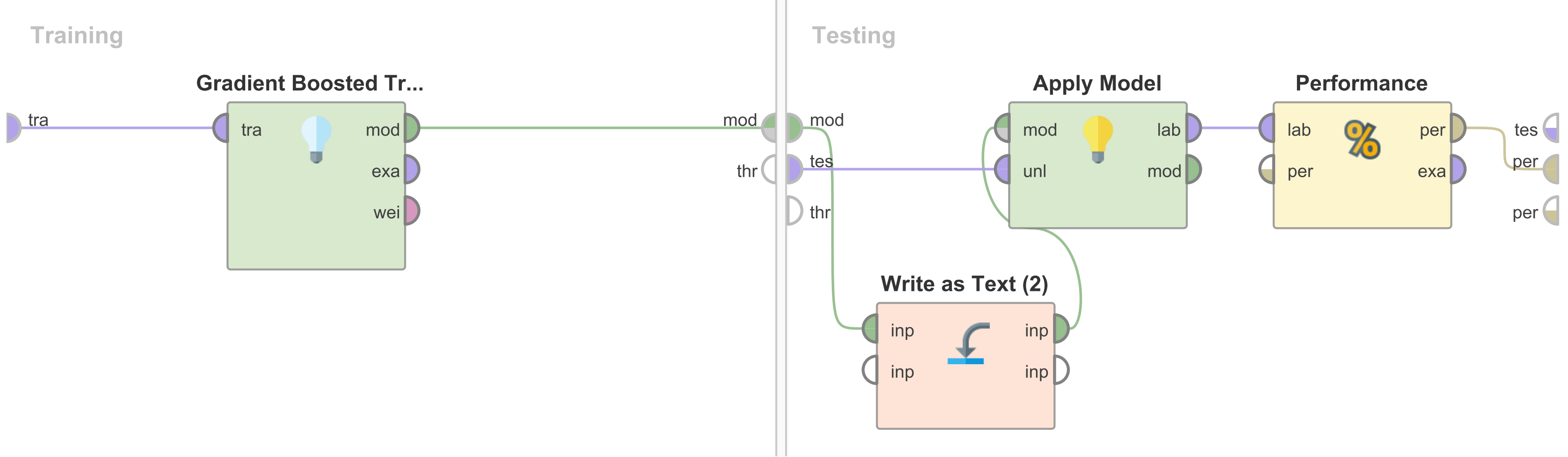

7) Random Forest + Weight by Tree Importance

Top Level

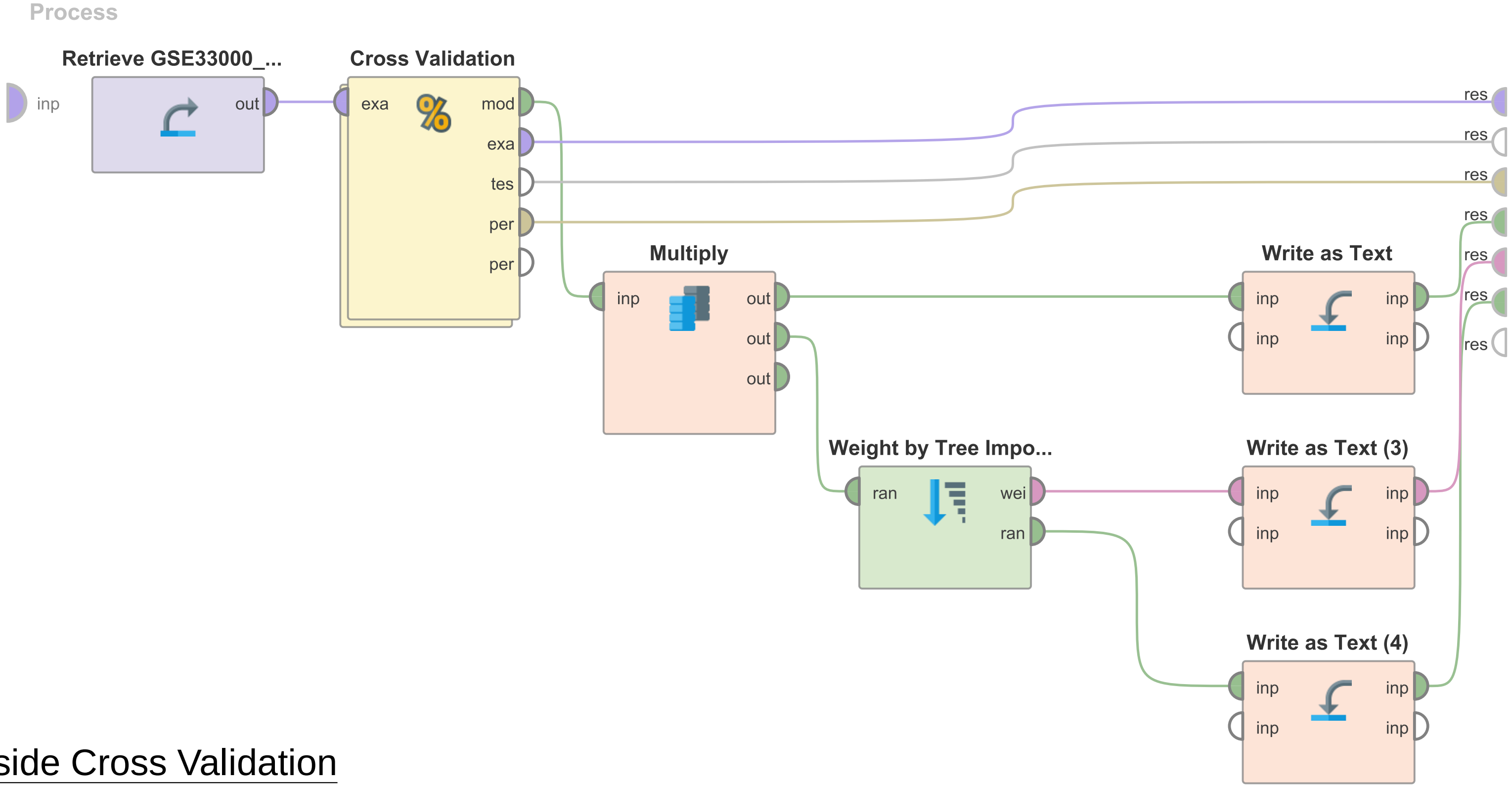

Inside Cross Validation

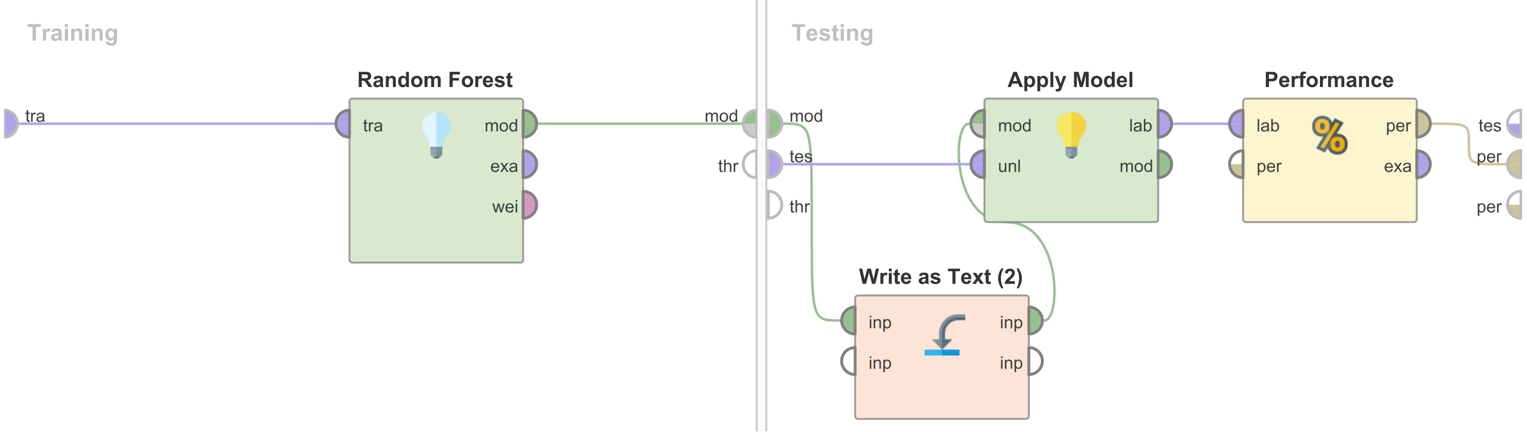

Supplement: Supplementary file 5 — Supplementary Information 5. [file 41598_2021_93085_MOESM5_ESM.pdf]
