# Supplementary figures and images for: Machine learning compensates fold-change method and highlights oxidative phosphorylation in the brain transcriptome of Alzheimer’s disease
Source: Sci Rep. 2021 Jul 1;11:13704. doi: 10.1038/s41598-021-93085-z (PMC8249453; doi:10.1038/s41598-021-93085-z)

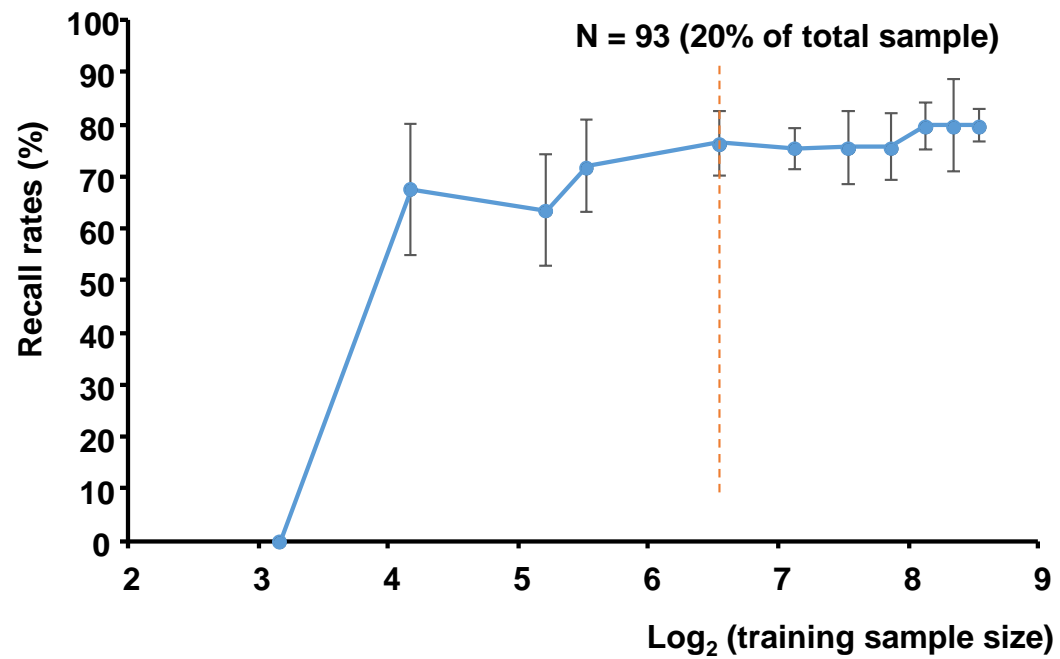

Supplement: Supplementary file 9 — Supplementary Information 9. [file 41598_2021_93085_MOESM9_ESM.pdf]
